# Molecular composition of meats: a novel look at microRNAs as functional nutrients

**DOI:** 10.1093/af/vfag005

**Published:** 2026-01-29

**Authors:** Amiton S de Mello, Nadini D H Gamage, Bradley S Ferguson, Tong Zhou

**Affiliations:** Department of Agriculture, Veterinary, and Rangeland Sciences, University of Nevada, Reno, NV; Department of Agriculture, Veterinary, and Rangeland Sciences, University of Nevada, Reno, NV; Department of Nutrition, University of Nevada, Reno, NV; Department of Physiology and Cell Biology, University of Nevada, Reno, NV

**Keywords:** meat, MicroRNA, nutrigenomics, transcriptomics

Implications

- Evidence suggests that microRNAs derived from animal-based foods may survive digestion and be absorbed by intestinal cells, potentially influencing host gene expression and metabolic regulation.
- Dietary microRNAs’ absorption and biological activity open new perspectives in nutrigenomics, highlighting how food components can modulate transcriptomic and metabolic responses in consumers.
- Understanding how animal nutrition affects tissue-specific microRNA expression supports the development of feeding strategies and gene-editing approaches to optimize both nutrient composition and bioactive RNA content in meat.
- The dual influence of diet and molecular regulation offers a pathway toward producing animal-derived foods with enhanced health-promoting properties while improving animal performance and product quality.

## Introduction

**Figure 1.**
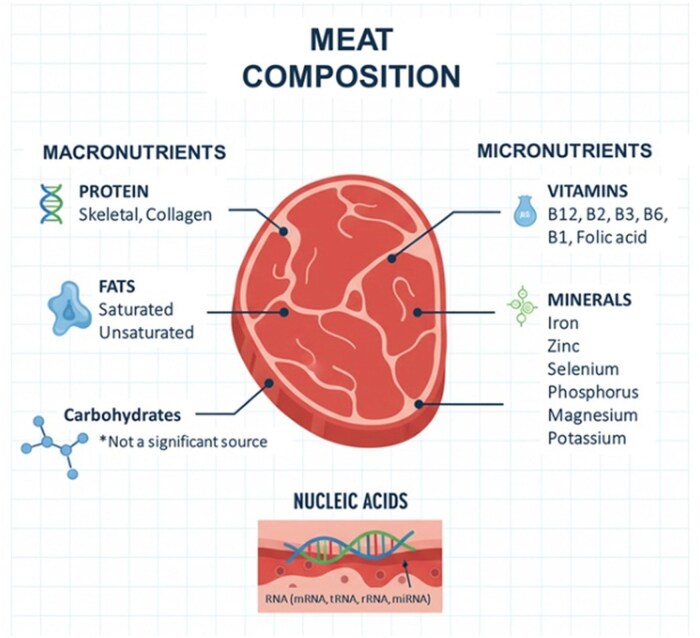
Comprehensive molecular and nutritional muscle tissue composition (Meat). *0 g per 1 Oz (85 g).

Meat provides a complex matrix of essential nutrients fundamental to human metabolism and physiological function (Figure 1). Proteins derived from meat are a rich source of indispensable amino acids that support muscle growth, maintenance, and repair by regulating protein synthesis and turnover (Wu, 2009). In addition to amino acids, meat supplies highly bioavailable minerals such as iron, zinc, and selenium, which are integral to oxygen transport, immune function, and antioxidant defense (Lombardi-Boccia et al., 2005). Meat is also a significant source of B-complex vitamins, particularly vitamin B12, niacin, and riboflavin. Those serve as coenzymes in energy metabolism and support mitochondrial activity and cellular homeostasis (Allen, 2009). Collectively, these nutrients underscore the central role of meat in sustaining metabolic health and neurodevelopment throughout the human lifespan.

While the roles of macro- and micronutrients have been extensively characterized, recent research has introduced the concept of food-derived “femtonutrients” (Gamage et al., 2025). Those biomolecules, such as microRNAs (miRNAs), may modulate gene expression and metabolic pathways. Meats are recognized as a source of ribonucleic acids (RNA), contributing to the body’s nucleotide pool (Sanderson, 1994). Notably, dietary ­miRNAs appear to be bioavailable, capable of gastrointestinal absorption, circulation, and interaction with host mRNAs, potentially altering protein synthesis and metabolic outcomes (Zhang et al., 2012; Baier et al., 2014). This recognition expands the nutritional role of meat beyond traditional macronutrients and micronutrients to include regulatory RNAs that may influence human physiology at the molecular level.

## MicroRNAs

**Figure 2.**
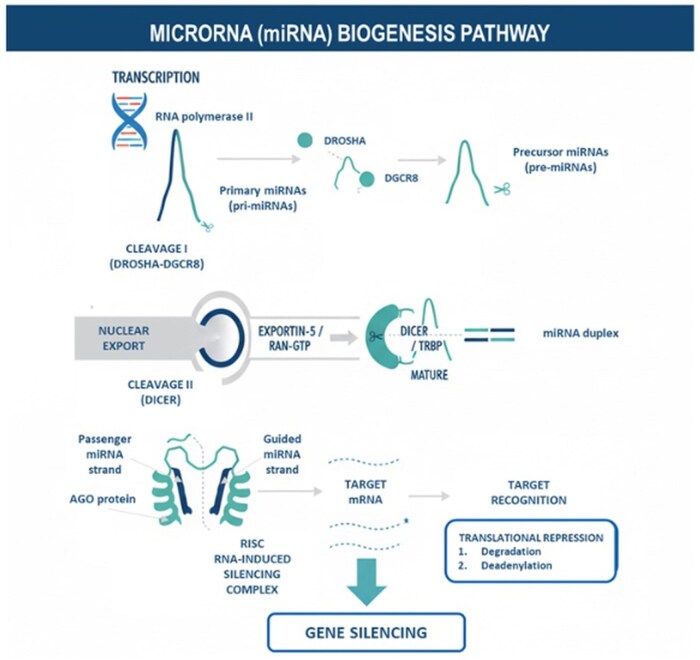
The canonical microRNA biogenesis pathway: from transcription to gene silencing.

MicroRNAs are a class of small, noncoding RNA molecules approximately 20 to 24 nucleotides in length that play essential roles in posttranscriptional regulation of gene expression. They fine-tune a wide range of biological processes, including development, differentiation, metabolism, and cellular homeostasis (Bartel, 2018; Gebert and MacRae, 2019). The biogenesis of miRNAs is a tightly coordinated, multistep process involving both nuclear and cytoplasmic enzymatic activities (Figure 2).

In the nucleus, miRNA genes are transcribed predominantly by RNA polymerase II, producing long primary transcripts known as primary miRNAs (pri-miRNAs). These pri-miRNAs contain characteristic stem-loop structures that serve as recognition motifs for the microprocessor complex, a multiprotein assembly composed of the RNase III enzyme Drosha and its cofactor DiGeorge syndrome critical region 8 (DGCR8). This complex cleaves the pri-miRNA to release a ∼70-nucleotide precursor miRNA (pre-miRNA), which retains the stem-loop structure essential for further processing (Ha and Kim, 2014).

The pre-miRNA is subsequently transported from the nucleus to the cytoplasm by the export receptor Exportin-5, which binds to pre-miRNAs in the presence of the protein Ran-GTP. This mechanism allows the complex to traverse the nuclear pore and release the cargo into the cytoplasm upon GTP hydrolysis (Görlich and Kutay, 1999). Once in the cytoplasm, the RNase III enzyme Dicer, assisted by the TRBP factor, cleaves the pre-miRNA near its terminal loop, generating a short double-stranded RNA duplex of approximately 22 nucleotides (Kim et al., 2009). This duplex contains two strands, the guide strand and the passenger strand. Both are temporarily associated with the Argonaute (AGO) proteins.

Subsequently, the passenger strand is degraded, leaving the guide strand incorporated into the AGO-containing effector complex. This complex is denominated RNA-induced silencing complex (RISC). The mature RISC complex recognizes target messenger RNAs (mRNAs) through partial or perfect sequence complementarity, typically within the 3′ untranslated region (3′ UTR). Binding of miRNA–RISC to the target mRNA results in translational repression or mRNA degradation, depending on the degree of complementarity (Jonas and Izaurralde, 2015; Bartel, 2018).

In mammals, miRNA-mediated silencing predominantly occurs through translational inhibition, mRNA deadenylation, and subsequent decapping and decay of target transcripts (Oliveto et al., 2017). Each miRNA can regulate multiple mRNA targets, creating a complex post-transcriptional regulatory network that integrates nutritional, hormonal, and environmental signals (Gebert and MacRae, 2019). This regulatory capacity underscores the emerging importance of miRNAs in nutrigenomics and their potential to modulate gene expression following the ingestion of animal-derived foods containing bioactive RNAs.

## Mechanisms of MicroRNA Regulation of Protein Synthesis

Incorporated into RISC, miRNAs recognize complementary sequences in the 3′ UTR of target mRNAs, guiding the complex to modulate protein production (Jonas and Izaurralde, 2015). They can reduce protein output by promoting mRNA degradation, often through recruitment of cellular decay machinery, or by inhibiting translation without affecting transcript stability. Translational repression may occur at multiple stages, including initiation, ribosome assembly, or elongation, fine-tuning the ­protein produced.

This precise regulation is essential for controlling growth, tissue development, and metabolic function in animal systems. miRNAs act as key modulators of processes such as cell differentiation, muscle development, adipogenesis, and nutrient metabolism. When miRNA regulation is disrupted, protein synthesis can become unbalanced, contributing to impaired growth, metabolic disorders, or tissue dysfunction. Understanding these regulatory mechanisms in livestock provides insight into how nutritional and environmental factors influence gene expression and animal performance (O’Brien et al., 2018).

## Availability of MicroRNAs in Beef

Research on the bioavailability of tissue-derived miRNAs in food remains limited. Most studies to date have focused on the role of miRNAs in livestock physiology, particularly their influence on growth, lipid metabolism, and meat tenderness (Duckett and Greene, 2022). Postmortem handling, including aging and cooking, is a key factor in determining miRNA stability. Skeletal muscle and adipose tissues are typically aged for 7 to 14 d before thermal processing. These treatments significantly influence miRNA abundance and persistence (Giotto et al. 2019; Gamage et al., 2025).

In fresh beef, miRNA expression in the *m. longissimus dorsi* varies according to anatomical location and intramuscular fat content. For example, miR-145, miR-143, miR-1246, and miR-23b-3p are highly expressed in intramuscular adipose tissue, whereas miR-2325c, miR-3616, and miR-2361 predominate in subcutaneous fat.

Aging also influences miRNA dynamics. Kakimoto et al. (2015) observed postmortem overexpression of specific miRNAs in cardiac tissue within 4 d of death, suggesting that similar processes occur in skeletal muscle. Giotto et al. (2019) reported a twofold increase in miR-19b, miR-23a, miR-24, and miR-206 after 14 d of postmortem aging. The increased expression of those miRNAs is aligned with the extent of tenderization determined by the activity of endogenous proteolytic systems, such as μ-calpain, during aging. Regarding cooking, thermal processing further modifies miRNA profiles. Dever et al. (2015) reported that cooking unaged beef reduced total miRNA content by 20 to 50%, though miR-10b-5p, miR-1, and miR-206 remained abundant.

Simulated gastrointestinal digestion of aged, sous-vide–cooked beef demonstrated that these miRNAs persisted through digestion, suggesting potential for intestinal absorption. Gamage et al. (2025) identified several bovine-specific miRNAs (e.g., bta-miR-2340, bta-miR-2440, bta-miR-2484, and bta-miR-11988) that remained stable post-digestion at concentrations comparable to physiologically absorptive levels. However, the functional interactions of these miRNAs with human mRNAs remain to be elucidated.

## Absorption and Intestinal Uptake of Dietary MicroRNAs

**Figure 3.**
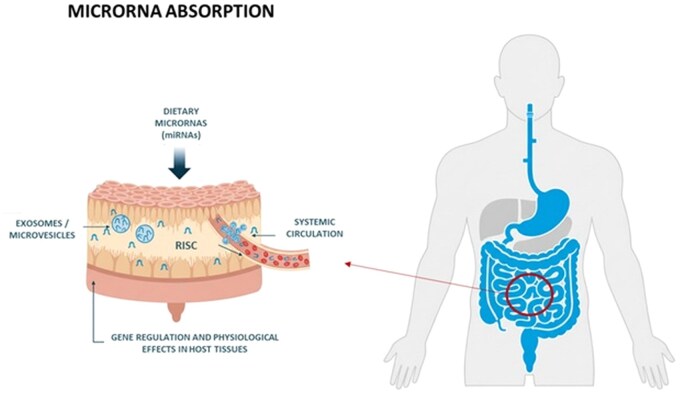
Absorption and intestinal uptake of dietary microRNAs.

Dietary miRNAs are selectively recognized by RNA-binding proteins, directed to multivesicular bodies, and packaged into intraluminal vesicles that are released as exosomes when the vesicles fuse with the cell membrane (Villarroya-Beltri et al., 2013). These vesicles protect RNA cargo from enzymatic degradation, enabling the transfer of small RNAs from food matrices to intestinal epithelial cells (Figure 3).

In the small intestine, enterocytes internalize exosomal miRNAs through endocytosis, membrane fusion, or receptor-mediated pathways involving scavenger receptors such as SR-BI and proteins including CD36 and Hsp70 (Wolf et al., 2015). Once internalized, exosomes release their RNA content into the cytoplasm, from which miRNAs can enter circulation either freely or complexed with Argonaute-2 (AGO2) or high-density lipoproteins (Vickers et al., 2011).

Animal and human studies support the intestinal uptake and systemic transport of dietary miRNAs. Baier et al. (2014) demonstrated that bovine milk-derived miR-29b and miR-200c were detectable in human plasma after milk consumption, while Izumi et al. (2015) showed that milk-derived exosomal miRNAs could cross intestinal epithelial monolayers in vitro. Similarly, animal studies confirmed that orally administered plant- or animal-derived miRNAs can be absorbed and delivered to peripheral tissues such as the liver and spleen (Zhang et al., 2012; Kusuma et al., 2016).

Although the efficiency of dietary miRNA absorption and their physiological impact remain debated, cumulative evidence supports their partial survival through digestion and entry into systemic circulation in bioactive forms. Food matrix composition, processing conditions, and vesicle integrity influence absorption (Gamage et al., 2025). The biological relevance of these exogenous miRNAs likely depends on complex interactions with endogenous RNA interference pathways.

## Nutrigenomic Modulation of Gene Expression in Skeletal Muscle

Nutrigenomics investigates the molecular interplay between dietary components and the genome, revealing how nutrients modulate gene expression, protein synthesis, and cellular metabolism in animal tissues. In livestock, nutritional inputs serve as metabolic signals that activate or repress key regulatory pathways such as the mammalian target of rapamycin (mTOR), AMP-­activated protein kinase (AMPK), and peroxisome proliferator-­activated receptors (PPARs). These pathways orchestrate the balance between anabolic and catabolic processes, thereby regulating muscle accretion, energy utilization, and tissue homeostasis (Baik et al., 2021). Nutrient sensing through these molecular hubs links feeding regimes directly to transcriptional and post-­transcriptional responses that shape growth performance, feed efficiency, and ultimately meat quality traits (Hocquette et al., 2012).

At the transcriptomic level, feeding strategies induce wide-ranging alterations in gene expression networks associated with myogenesis, lipid metabolism, and oxidative capacity of skeletal muscle. Nutrient composition, particularly dietary energy density, amino acid balance, and fatty acid profile, has been shown to influence the expression of both structural and regulatory genes, including *MYOD1*, *MYOG*, *FABP4*, and *PPARγ*, which are essential for muscle fiber differentiation and lipid partitioning (Yan et al., 2023). Concurrently, the expression of muscle-enriched miRNAs such as miR-1, miR-133, and miR-206 responds sensitively to dietary modulation, acting as post-transcriptional regulators of gene networks governing muscle development and metabolic adaptation.

These nutrigenomic responses extend beyond gene-level regulation to encompass broader cellular functions, including mitochondrial biogenesis, oxidative phosphorylation, and the transition between glycolytic and oxidative muscle fibers. Through the integration of transcriptomic, proteomic, and metabolomic data, it is possible to identify molecular biomarkers that reflect nutritional status and predict muscle growth potential. Such insights support the development of precision nutrition strategies designed to optimize feed utilization, enhance carcass composition, and promote sustainable animal production. Collectively, the nutrigenomic modulation of skeletal muscle underscores the central role of diet in orchestrating molecular pathways that define both ­productive performance and meat quality in livestock species.

## Animal Diets Affect MicroRNA Expression in Edible Tissues

**Figure 4.**
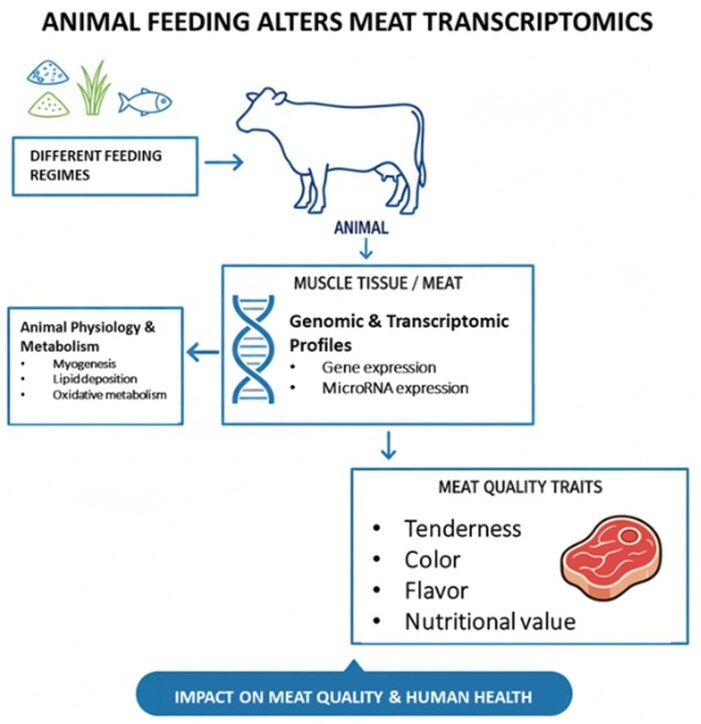
Conceptual diagram illustrating how animal feeding influences meat quality traits, nutritional composition, and transcriptomic profiles.

Animal diet composition profoundly influences the expression profile of miRNAs in tissues that ultimately serve as food sources, such as skeletal muscle, liver, and adipose tissue ­(Figure 4). Nutrients act as signaling molecules that interact with transcriptional regulators and metabolic pathways, modulating miRNA biogenesis and expression. Dietary macronutrients, including proteins, lipids, and carbohydrates, regulate miRNA transcription and maturation (Zhou et al., 2019). For example, feeding high-fat diets to livestock has been shown to modify the expression of miRNAs associated with lipid metabolism, such as *miR-122*, *miR-33*, and *miR-143*, which target genes involved in fatty acid oxidation and adipogenesis (Jin et al., 2010). Similarly, protein supplementation or amino acid imbalance can affect the expression of muscle-specific miRNAs (myomiRs), including *miR-1*, *miR-133a*, and *miR-206*, which regulate muscle growth, satellite cell differentiation, and postmortem proteolytic activity.

In ruminant production systems, the inclusion of byproducts such as distillers’ grains or oilseed meals has been reported to alter both tissue lipid composition and the expression of miRNAs governing lipid transport and energy utilization (Ma et al., 2023). Nutritional interventions that enhance antioxidant or anti-inflammatory capacity, such as dietary supplementation with polyphenols, selenium, or omega-3 fatty acids, can also reshape the miRNA landscape, influencing stress response and meat quality traits (Wang et al., 2021). These nutrigenomic interactions suggest that feed formulation strategies can indirectly shape the molecular composition of animal-derived foods, potentially modifying the abundance and stability of bioactive miRNAs that persist through processing and are later ingested by humans. Understanding these diet–miRNA relationships provides an innovative framework for optimizing both animal performance and the functional quality of meat as a source of bioactive molecules.

## Conclusion

MicroRNAs are key post-transcriptional regulators that modulate gene expression through mRNA silencing, fine-tuning protein synthesis, and cellular metabolism. Emerging research suggests that dietary miRNAs can persist through gastrointestinal conditions, be absorbed by the intestinal epithelium, and potentially influence host gene expression. However, the mechanisms and physiological significance of these interactions remain to be elucidated.

Nutritional management clearly influences the transcriptomic and miRNA landscapes of animal tissues, leading to new hypotheses about how feeding strategies might shape the molecular composition of animal-derived foods. As gene editing technologies become increasingly integrated into livestock production, combining genomic and nutritional management offers a powerful approach to optimizing meat’s nutrient and transcriptomic profiles. This integration represents a promising frontier for producing animal-source foods that promote efficiency, quality, and potential consumer health benefits.

## Author Biographies

Dr. Amilton de Mello is the Endowed Professor of Meat Science and Muscle Biology at the ­University of Nevada, Reno. He obtained his D.V.M. from the University of Marilia, his M.S. from São Paulo State University, and his Ph.D. from the University of Nebraska, Lincoln. His research focuses on the interface between animal nutrition, muscle biology, and meat science, ­aiming to improve sustainability across livestock production systems and public health. Dr. de Mello leads an integrated research program investigating how pre and postharvest factors influence the molecular composition of skeletal muscle and how meat nutrigenomics affects human metabolism. He has authored numerous peer-reviewed publications and collaborates extensively with national and international partners in academia and industry. He is deeply committed to teaching and mentoring the next generation of meat scientists, emphasizing applied innovation and translational research to support global food security and consumer health.

Nadini Haththotuwe Gamage is a Ph.D. candidate in the Department of Animal and Rangeland Sciences at the University of Nevada, Reno. She holds a master’s degree in Chemistry, with a background in molecular biology. Her doctoral research focuses on understanding dietary small RNAs’ absorption kinetics and functional roles, particularly those derived from beef, and their potential nutrigenomic effects on mammalian health. She employs molecular biology techniques and RNA sequencing to explore how diet-derived bioactive molecules regulate gene expression, protein synthesis, and metabolic pathways. Her future research interests will focus on how dietary bioactive molecules regulate gene expression, protein function, and metabolic pathways to promote health or drive disease.

Dr. Brad Ferguson is an Associate Professor of Nutrition at the University of Nevada, Reno, where his research focuses on the intersection of nutrition, metabolism, and human health. His work explores how dietary components influence metabolic pathways, inflammation, and chronic disease risk, with a particular emphasis on the molecular and physiological mechanisms linking nutrition to cardiometabolic health. Dr. Ferguson’s research examined the role of histone deacetylase (HDAC) enzymes in regulating cardiac dysfunction and heart failure. His research also showed that loss-of-HDAC6 improved cardiac systolic function and reduced skeletal muscle atrophy/wasting in response to angiotensin II-induced hypertension. His current work examines the role of sarcomere protein acetylation in muscle contractility and relaxation; an emphasis is placed upon understanding how acetylation of skeletal muscle alpha-actin (ACTA1) regulates the biophysical interactions between actin-myosin and actin-tropomyosin in cardiac and skeletal muscle. His contributions continue to enhance understanding of cardiac metabolic regulation and human well-being.

**Dr. Tong Zhou** is an Associate Professor at the Department of Physiology and Cell Biology, University of Nevada, Reno. He earned his doctoral degree from Southeast University, along with postdoctoral training at the University of Texas, Austin. His broad academic and research background has been rooted in bioinformatics and computational biology. He has a long-standing interest in translational bioinformatics and computational RNA biology, including developing and using computational tools and bioinformatics software to analyze high-throughput omics data and understanding the “RNA code” that contributes to epigenetic inheritance.

## References

[vfag005-B1] Allen L.H. 2009. How common is vitamin B-12 deficiency? Am. J. Clin. Nutr. 89(2):693S–696S. doi:10.3945/ajcn.2008.26947a 19116323

[vfag005-B2] Baier S.R. , Nguyen C., Xie F., Wood J.R., Zempleni J. 2014. MicroRNAs are absorbed in biologically meaningful amounts from nutritionally relevant doses of cow milk and affect gene expression in peripheral blood mononuclear cells, HEK-293 kidney cell cultures, and mouse livers. J. Nutr. 144(10):1495–1500. doi:10.3945/jn.114.196436 25122645 PMC4162473

[vfag005-B3] Baik M. , Park S.J., Na S.W., Piao M.Y. 2021. Nutrigenomics in livestock: the potential roles of nutrients in regulating animal growth and metabolism. Anim. Nutr. 7(2):480–489. doi:10.1016/j.aninu.2021.02.002

[vfag005-B4] Bartel D.P. 2018. Metazoan microRNAs. Cell. 173(1):20–51. doi:10.1016/j.cell.2018.03.006 29570994 PMC6091663

[vfag005-B0830346] Dever J. T. , Kemp M. Q., Thompson A. L., Keller H. G. K., Waksmonski J. C., Scholl C. D., and Barnes D. M. 2015. Survival and diversity of human homologous dietary micrornas in conventionally cooked top sirloin and dried bovine tissue extracts. PLoS ONE. 10(9):e0138275. doi:10.1371/journal.pone.0138275 26394052 PMC4578893

[vfag005-B6] Duckett S. K , and Greene M. A. 2022. Identification of microrna transcriptome involved in bovine intramuscular fat deposition. Front. Vet. Sci. 9:883295. doi:10.3389/fvets.2022.883295 35498736 PMC9051433

[vfag005-B7] Gamage N.D.H. , Ferguson B.S., de Mello A.S. 2025. Absolute quantification of bovine-specific microRNAs uncovers their differential stability during aging, cooking, and simulated digestion. Amer. Meat Sci. Assoc. https://meatscience.org/docs/default-source/publications-resources/rmc/rmc-technical-abstracts.pdf? sfvrsn=1487baeb_1

[vfag005-B8] Gebert L.F. , MacRae I.J. 2019. Regulation of microRNA function in animals. Nat. Rev. Mol. Cell. Biol. 20(1):21–37. doi:10.1038/s41580-018-0045-7 30108335 PMC6546304

[vfag005-B9] Giotto F.M. , Evans L.W., Ferguson B.S., de Mello A.S. 2019. Availability of human homologous dietary microRNAs in cooked beef. Int. Cong. Meat Sci. Tech. https://digicomst.ie/wp-content/uploads/2020/05/2019_16_31-1.pdf

[vfag005-B10] Görlich D. , Kutay U. 1999. Transport between the cell nucleus and the cytoplasm. Annu. Rev. Cell. Dev. Biol. 15:607–660. doi:10.1146/annurev.cellbio.15.1.607 10611974

[vfag005-B11] Ha M. , Kim V.N. 2014. Regulation of microRNA biogenesis. Nat. Rev. Mol. Cell. Biol. 15(8):509–524. doi:10.1038/nrm3838 25027649

[vfag005-B12] Hocquette J.F. , Ortigues-Marty I., Pethick D.W., Herpin P., Fernandez X. 2012. Nutritional and hormonal regulation of energy metabolism in skeletal muscles of meat-producing animals. Livest. Sci. 150(2–3):199–210. doi:10.1016/j.livsci.2012.09.004

[vfag005-B13] Izumi H. , Tsuda M., Sato Y., Kosaka N., Ochiya T., Iwamoto H., Namba K., Takeda Y. 2015. Bovine milk exosomes contain microRNA and mRNA and are taken up by human macrophages. J. Dairy. Sci. 98(5):2920–2933. doi:10.3168/jds.2014-8760 25726110

[vfag005-B723222205] Jin W. , Dodson M. V., Moore S. S., Basarab J. A., and Guan L. L. 2010. Characterization of microrna expression in bovine adipose tissues: A potential regulatory mechanism of subcutaneous adipose tissue development. BMC Molecular Biol. 11(1). doi:10.1186/1471-2199-11-29 PMC2874793 20423511

[vfag005-B15] Jonas S. , Izaurralde E. 2015. Towards a molecular understanding of microRNA-mediated gene silencing. Nat. Rev. Genet. 16(7):421–433. doi:10.1038/nrg3965 26077373

[vfag005-B745712890] Kakimoto Yu. , Kamiguchi H., Ochiai E., Satoh F., and Osawa M. 2015. MicroRNA stability in postmortem FFPE tissues: Quantitative analysis using autoptic samples from acute myocardial infarction patients. PLoS ONE. 10(*6*):e0129338. doi:10.1371/journal.pone.0129338 26046358 PMC4457786

[vfag005-B16] Kim V.N. , Han J., Siomi M.C. 2009. Biogenesis of small RNAs in animals. Nat. Rev. Mol. Cell. Biol. 10(2):126–139. doi:10.1038/nrm2632 19165215

[vfag005-B17] Kusuma R.J. , Manca S., Friemel T., Sukreet S., Nguyen C., Zempleni J. 2016. Human vascular endothelial cells transport foreign exosomes from cow’s milk by endocytosis. Am. J. Physiol. Cell. Physiol. 310(10):C800–C807. doi:10.1152/ajpcell.00389.2015 26984735 PMC4895447

[vfag005-B20] Lombardi-Boccia G. , Lanzi S., Aguzzi A. 2005. Aspects of meat quality: trace elements and B vitamins in raw and cooked meats. J. Food. Compost. Anal. 18(1):39–46. doi:10.1016/j.jfca.2004.02.003

[vfag005-B9509463] Ma Z. , Wang C., Wang Bo., Yao L., Kong B., Shan A., Li J., and Meng Q. 2023. Effects of feeding corn distillers dried grains with solubles on muscle quality traits and lipidomics profiling of finishing pigs. Animals. 13(24):3848. doi:10.3390/ ani13243848 38136885 10.3390/ani13243848 PMC10741057

[vfag005-B21] O’Brien J. , Hayder H., Zayed Y., Peng C. 2018. Overview of microRNA biogenesis, mechanisms of action, and circulation. Front. Endocrinol. ­(Lausanne). 9:402. doi:10.3389/fendo.2018.00402 PMC6085463 30123182

[vfag005-B23] Oliveto S. , Mancino M., Manfrini N., Biffo S. 2017. Role of microRNAs in translation regulation and cancer. World J. Biol. Chem. 8(1):45–56. doi:10.4331/wjbc.v8.i1.45 28289518 PMC5329714

[vfag005-B4844997] Sanderson I. R. , and He Y. 1994. Nucleotides and intestinal development and repair. J. Nutr. 124(8 Suppl):1419S–1422S. 10.1093/jn/124.suppl_8.1436S 8064399

[vfag005-B24] Vickers K.C. , Palmisano B.T., Shoucri B.M., Shamburek R.D., Remaley A.T. 2011. MicroRNAs are transported in plasma and delivered to recipient cells by high-density lipoproteins. Nat. Cell. Biol. 13(4):423–433. doi:10.1038/ncb2210 21423178 PMC3074610

[vfag005-B25] Villarroya-Beltri C. , Gutiérrez-Vázquez C., Sánchez-Cabo F., Pérez-Hernández D., Vázquez J., Martin-Cofreces N., Martinez-Herrera D.J., Pascual-­Montano A., Mittelbrunn M., Sánchez-Madrid F. et al. 2013. Sumoylated hnRNPA2B1 controls the sorting of miRNAs into exosomes through binding to specific motifs. Nat. Commun. 4:2980. doi:10.1038/ncomms3980 24356509 PMC3905700

[vfag005-B26] Wang X. , Yang C., Li S. 2021. Selenium supplementation modulates hepatic and muscular microRNAs associated with oxidative stress and inflammation in broilers. Poult. Sci. 100(5):101017. doi:10.1016/j.psj.2021.101017 33652521 PMC7936184

[vfag005-B28] Wolf T. , Baier S.R., Nguyen C., Zempleni J. 2015. Uptake of milk-derived exosomes by murine macrophages. J. Nutr. Biochem. 26(11):1113–1119. doi:10.1016/j.jnutbio.2015.05.004

[vfag005-B29] Wu G. 2009. Amino acids: metabolism, functions, and nutrition. Amino Acids. 37(1):1–17. doi:10.1007/s00726-009-0269-0 19301095

[vfag005-B68778903] Yan E. , Guo J., and Yin J. 2023. Nutritional regulation of skeletal muscle energy metabolism, lipid accumulation and meat quality in pigs. Animal Nutrition. 14:185–192. doi:10.1016/j.aninu.2023.04.009 37808951 PMC10556049

[vfag005-B30] Zhang L. , Hou D., Chen X., Li D., Zhu L., Zhang Y., Li J., Bian Z., Liang X., Cai X. et al. 2012. Exogenous plant microRNAs function in mammalian cells to regulate gene expression. Cell. Res. 22(1):107–126. doi:10.1038/cr.2011.158 21931358 PMC3351925

[vfag005-B32] Zhou Y. , Liu X., Zhang X. 2019. Nutrient signaling pathways and microRNA-mediated regulation in metabolic tissues. Trends Endocrinol. Metab. 30(9):703–715. doi:10.1016/j.tem.2019.06.004

